# Assessment of variations in air quality in cities of Ecuador in relation to the lockdown due to the COVID-19 pandemic

**DOI:** 10.1016/j.heliyon.2023.e17033

**Published:** 2023-06-12

**Authors:** Oliva Atiaga, Fernanda Guerrero, Fernando Páez, Rafael Castro, Edison Collahuazo, Luís Miguel Nunes, Marcelo Grijalva, Iván Grijalva, Xosé Luis Otero

**Affiliations:** a Departamento de Ciencias de la Tierra y la Construcción, Universidad de las Fuerzas Armadas ESPE, Av. General Rumiñahui s/n, Sangolquí, P.O. Box 171-5-231B, Ecuador; b CRETUS. Departamento de Edafoloxía e Química Agrícola, Facultade de Bioloxía, Universidade de Santiago de Compostela, Campus Sur, 15782 Santiago de Compostela, Spain; c Geospace Solutions, Av. Manuel Córdova Galarza km 4.5, P.O. Box 170177, Ecuador; d Faculdade de Ciências e Tecnologia, Universidade do Algarve, Campus de Gambelas, Faro, Portugal; e CERIS, Instituto Superior Técnico, Universidade de Lisboa, Av. Rovisco Pais 1, 1049-001, Lisboa, Portugal; f Departamento de Ciencias de la Vida, Universidad de las Fuerzas Armadas ESPE, Av. General Rumiñahui s/n, Sangolquí, P.O. Box 171-5-231B, Ecuador; g Independent consultant. Avenida Amazonas N22-62 y Ramirez Dávalos, PO BOX 170526, Quito, Ecuador; h REBUSC Network of Biological Field Stations of the University of Santiago de Compostela, Marine Biology Stations of A Graña and Ferrol, University of Santiago de Compostela, 15782 Santiago de Compostela, Spain

**Keywords:** Air quality, Covid-19, Nitrogen dioxide, Sulfur dioxide, Ozone

## Abstract

This study analyzes the effect of lockdown due to COVID-19 on the spatiotemporal variability of ozone (O_3_), sulfur dioxide (SO_2_), and nitrogen dioxide (NO_2_) concentrations in different provinces of continental Ecuador using satellite information from Sentinel – 5P. The statistical analysis includes data from 2018 to March 2021 and was performed based on three periods defined a priori: before, during, and after lockdown due to COVID-19, focusing on the provinces with the highest concentrations of the studied gases (hotspots). The results showed a significant decrease in NO_2_ concentrations during the COVID-19 lockdown period in all the study areas: the Metropolitan District of Quito (DMQ) and the provinces of Guayas and Santo Domingo de los Tsáchilas. In the period after lockdown, NO_2_ concentrations increased by over 20% when compared to the pre-lockdown period, which may be attributable to a shift towards private transportation due to health concerns. On the other hand, SO_2_ concentrations during the lockdown period showed irregular, non-significant variations; however, increases were observed in the provinces of Chimborazo, Guayas, Santa Elena, and Morona Santiago, which could be partly attributed to the eruptive activity of the Sangay volcano during 2019–2020. Conversely, O_3_ concentrations increased by 2–3% in the study areas; this anomalous behavior could be attributed to decreased levels of NO_x_, which react with ozone, reducing its concentration. Finally, satellite data validation using the corresponding data from monitoring stations in the DMQ showed correlation values of 0.9 for O_3_ data and 0.7 for NO_2_ data, while no significant correlation was found for SO_2_.

## 1 Introduction

Air pollution is among the most challenging environmental issues needing to be addressed at the local, regional, and global scales [1]. The available evidence suggests that short- and long-term exposure to environmental air pollution is associated with negative health outcomes both in developed and in developing countries [2,3]. Over 90% of the world's population is exposed to harmful levels of air pollutants, exceeding the thresholds set by the World Health Organization [4], which is considered one of the leading causes of early mortality and morbidity worldwide [5]. Thus, in 2021, a total of 3.7 million deaths were attributed to air pollution. These deaths took place primarily in low- and middle-income countries, which account for 82% of the world's population [4].

Atmospheric pollution primarily leads to the development of respiratory and cardiovascular diseases [1,2,5], which particularly affect elderly people and children, due to their higher vulnerability, and generally marginalized social groups, due to their longer exposure times to low-quality environments [2,4].

In recent decades, air quality has substantially declined due to the increasing emissions of pollutants into the atmosphere, such as sulfur dioxide (SO_2_), nitrogen oxides (NOx), particulate matter (PM), volatile organic compounds (VOCs), and carbon monoxide (CO), as well as to the increase in secondary pollutants formed in the atmosphere, such as ozone (O_3_), which is generated from nitrogen oxides and hydrocarbons present in the atmosphere under UV exposure [5].

In Ecuador, a country located in South America, air quality is one of the most relevant environmental issues, particularly in large cities such as Quito [6], a city located in the Andes mountains, characterized by a complex topography (with a mean altitude of 2800 m asl), and with raised areas that constitute a natural barrier that limits pollutant dispersal [7]. Low air quality is also attributed to the use of low-quality fuels and to increased road traffic [[7], [8], [9]].

When the World Health Organization declared the global pandemic caused by the Covid-19 virus, Ecuador established a series of lockdown measures to prevent community transmission, as did most countries in the world. Among the measures implemented in Ecuador were restrictions to vehicle and pedestrian mobility and suspension of on-site work in both the public and private sectors (COE, 2020a); these measures had an impact on air quality [11,12]. Hard lockdown measures were implemented on March 17, 2020 [10] and were gradually lifted from May 20, 2020 [13].

Previous studies have focused on determining spatial variations in concentrations, especially of NO_2_, during lockdown periods in cities in different countries around the world, such as China, India, Spain, Italy, and Ecuador [12,[14], [15], [16], [17]]. In Ecuador, variations in ozone concentrations during this period were analyzed based on data from one air quality monitoring station located in Cumbaya-Ecuador [11].

On the other hand, in contrast with traditional air pollution monitoring technologies, rapidly developing atmospheric monitoring methods via satellite remote sensing have gradually become critical technical tools for atmospheric monitoring worldwide [14], as they allow for low-cost real-time data collection. However, these methods have some limitations, especially regarding the fact that they use column density data instead of surface or near-surface concentrations [18].

For the aforementioned reasons, this study conducted a spatiotemporal analysis of trace gases such as NO_2_, O_3_, and SO_2_ to determine their variations as a function of the pandemic-related lockdown in the Metropolitan District of Quito and in the Ecuadorian provinces with the highest concentration values (hotspots). Data were obtained from Sentinel-5P TROPOMI and were processed using the software ENVI 5.5.6 to analyze how the decisions made at the beginning of the pandemic affected air quality.

## 2 Methodology

### 2.1 Data collection and processing

In this study we used offline data of level 3 of NO2, SO2 and O3 [mol/m^2^], obtained from the sensor TROPOMI (Tropospheric Monitoring Instrument) that is on board the satellite Sentinel-5P [[19], [20], [21]]. Monthly mean was calculated for continental Ecuador from December 2018 to October 2021, through Google Earth Engine (GEE) [22]. All of the S5P datasets have three versions: Near Real-Time (NRTI) and Offline (OFFL). NRTI data are available within 3 h after data acquisition whereas OFFL data are available within a few days after acquisition [23]. For this work, we employed the collections Sentinel-5P OFFL NO2 - Offline Nitrogen Dioxide, Sentinel-5P OFFL SO2 - Offline Sulfur Dioxide and Sentinel-5P OFFL O3 - Offline Ozone, of which more information is detailed in Table 1. The data quality filter was carried out through the *harpconvert* tool with the *binspatial* operation [24], which filters and removes pixels that do not comply with the quality guarantee (*qa*-value) to remove cloud contamination and other poor-quality retrievals. Specifically, pixels with a *qa*-value <75% were filtered for the band *tropospheric NO2 column number density* and with a qa-value <50% for the band *SO2 column number density*. On the other hand, the *qa*-value for L3 O3 product is adjusted before running *harpconvert* to satisfy all of the following criteria: ozone_total_vertical_column in [0, 0.45]; ozone effective temperature in [180, 260]; ring scale factor in [0, 0.15]; effective_albedo in [−0.5, 1.5].

**Table 1:** Imagery collections and selected bands.

Image name	Band name	Description	Resolution (m)	Units
COPERNICUS/S5P/OFFL/L3_NO2	tropospheric_NO2_column_number_density	tropospheric vertical column density	1113.2	mol/m^2^
COPERNICUS/S5P/OFFL/L3_SO2	SO2_column_number_density	SO2 vertical column density at ground level	1113.2	mol/m^2^
COPERNICUS/S5P/OFFL/L3_O3	O3_column_number_density	Total atmospheric column ozone concentration	1113.2	mol/m^2^
COPERNICUS/S5P/OFFL/L3_O3_TCL	Ozone_tropospheric_mixing_ratio	Average tropospheric ozone	111320 y resolution 55660	mol/m^2^

Additionally, the municipality of Quito has an atmospheric monitoring network, designed according to the recommendations of the US-Environmental Protection Agency [7,9,25], from the eight monitoring stations (Fig. 7 in SM) belonging to such a network (REMMAQ): Belisario, Carapungo, Centro Histórico, Cotocollao, El Camal, Guamaní, Los Chillos, and Tumbaco, monthly mean concentrations (mg/m^3^) were obtained for trace gases NO_2_ and SO_2_; as for O_3_, the San Antonio de Pichincha station was also included. The characteristics and description of the equipment and methods of the REMMAQ network is provided in Supplementary Material (Table 1 SM).

Daily data were obtained for the study period, between December 2018 and March 2021; the data corresponded to the time lapse between 13:00 and 14:00 every day, since satellite Sentinel-5P goes over Ecuador approximately at 13:30 local time [26]. The network follows international standards for measurement and quality control (details about equipment and measured parameters may be found here: http://www.quitoambiente.gob.ec/index.php/generalidades).

Satellite data corresponding to the geographic coordinates of the different monitoring stations were extracted from satellite images, and monthly mean data were generated using the appropriate ArcGIS 10.8 tool (extract multi values to points), which allowed extracting cell values for specific locations.

Based on level-3 data for NO_2_, SO_2_, and O_3_, satellite images collected by Sentinel- 5P were processed and subsequently georeferenced to a global spatial system, and time series were generated using the scientific software ENVI 5.5.6, which allowed generating monthly mean images.

The generated images were used to determine the areas with the highest concentrations (hotspots); for this purpose, a spatiotemporal statistical analysis was performed by applying Anselin Local Moran's I statistic, the best known and most widely used spatial assessment statistic for this purpose [14,27]. Based on the satellite images and the hotspots determined, the provinces of Ecuador with the highest concentrations of the studied gases were selected. Moreover, the Metropolitan District of Quito (DMQ) was selected for the spatiotemporal analysis of the concentrations of the studied gases; this district includes the urban area of Quito and neighboring rural municipalities. A network of rectangular cells was then generated in ArcGIS to determine the atmospheric sampling points and to extract data at a distance of 5.5 km between points, this due to the pixel size of the image, covering the whole area of Continental Ecuador. In addition to monthly data analysis, study periods were established before (December 2018–February 2020), during (March 2020–May 2020), and after lockdown (June 2020–March 2021).

Data were then statistically analyzed and validated. For this purpose, level-3 data were verified against data from the Metropolitan District of Quito's Atmospheric Monitoring Network (REMAQ) during the same study period and in the same locations as the monitoring stations. Additionally, trends were compared between level-3 data for tropospheric column ozone and O_3_ total column density. Moreover, in order to validate satellite data, the relationship between level-3 satellite data for the three studied gases and the corresponding data from the DMQ monitoring stations was analyzed by least-squares fitting of linear models with the former as dependent variables. Analysis of variance [28] was used to identify significant differences between pre-lockdown, lockdown, and post-lockdown periods per region.

## 3 Results

**Fig. 1:**
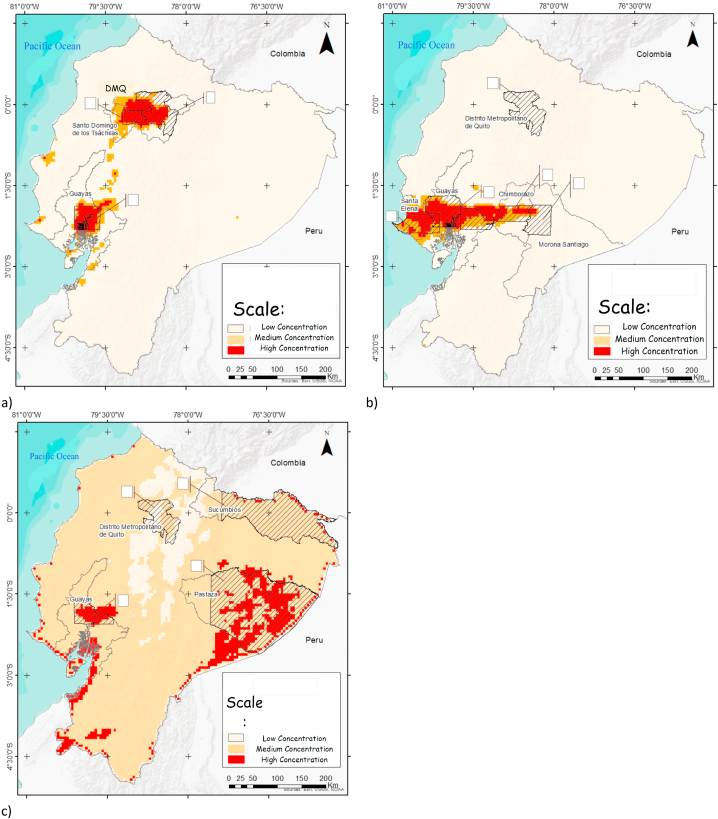
a) NO_2_ concentrations; b) SO_2_ concentrations; c) O_3_ concentrations in continental Ecuador provinces.

According to the determination of tropospheric vertical column density (TVCD) hotspots (Fig. 1a–c) and to the spatio-temporal analysis (Figs. 1 to 3 in Supplementary Material), the provinces and the number of data selected for NO_2_ were: Metropolitan District of Quito (DMQ) (n = 142), Guayas (n = 68), and Santo Domingo (n = 37). Regarding the analysis for sulfur dioxide, Santo Domingo was replaced by Morona Santiago (n = 71), Chimborazo (n = 40), and Santa Elena (N = 73); in Guayas, the area of the SO_2_ hotspot was larger than the one for NO_2_ and thus required a higher number of locations (n = 187). Finally, for O_3,_ the two large urban areas DMQ (n = 142) and Guayas (n = 45) remained, and two additional forest provinces were included: Pastanza (n = 389) and Sucumbios (n = 194). The total number of records per province, including monthly data, was the product of the number of locations by the monthly records in the time series (December 2018–March 2021).

Comparing the different regions, NO_2_ concentrations were significantly lower in DQM (p < 0.05) than in the provinces of Guayas and Santo Domingo de los Tsáchilas. On the other hand, in Guayas, NO_2_ was observed to reach significantly higher pre-lockdown concentrations (p < 0.05) than in the province of Santo Domingo de los Tsáchilas. However, when comparing NO_2_ concentrations during and after lockdown, the provinces of Guayas and Santo Domingo de los Tsáchilas did not show any significant variations (p > 0.05) (Table 2).

**Table 2:** Trace gas densities in provinces of Ecuador during the periods.

Province	Before Lockdown	Lockdown	After Lockdown	% change post to pre lockdown
**Nitrogen dioxide (μmol/m**^**2**^**)**				
Santo Domingo de los Tsáchilas Province n = 1369	48 ± 49	48 ± 49	55 ± 78	+14%*
Guayas Province n = 2516	54 ± 12	50 ± 80	62 ± 13	+15%*
DMQ n = 5254	42 ± 57	40 ± 52	48 ± 78	+19%*
**Sulfur dioxide (μmol/m**^**2**^**)**				
DMQ n = 5254	31 ± 53	34 ± 60	34 ± 84	+9.7*
Guayas Province n = 6919	11 ± 18	21 ± 20	48 ± 40	+380%*
Santa Elena Province n = 2701	12 ± 14	19 ± 45	42 ± 45	+250%*
Morona Santiago Province **n = 2627**	11 ± 23	12 ± 20	29 ± 130	+164%*
Chimborazo Province n = 1480	13 ± 19	31 ± 33	40 ± 84	+208%*
**Ozone (mmol/m**^**2**^**)**				
DMQ n = 5254	113 ± 0.3	110 ± 0.1	112 ± 0.4	−01%**
Guayas Province n = 1665	115 ± 0.3	112 ± 0.1	114 ± 0.4	−08%**
Pastaza Province n = 14393	115 ± 0.3	111 ± 0.1	114 ± 0.4	−08%**
Sucumbios Province n = 7178	115 ± 0.3	112 ± 0.1	114 ± 0.4	−08%**

*: Significant difference; **: non-significant difference See text for details.

**Fig. 2:**
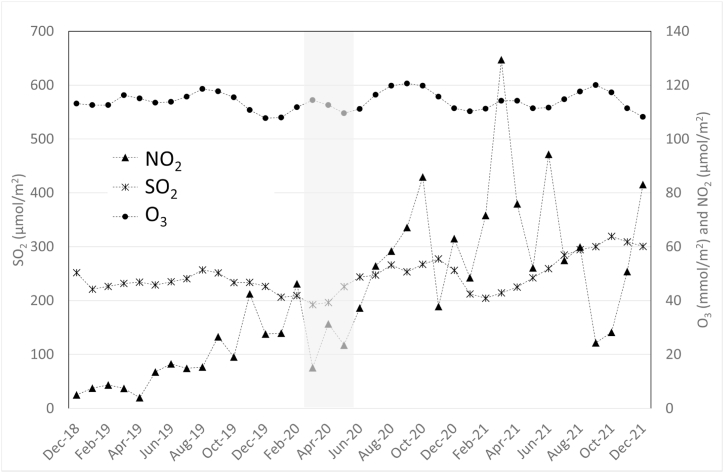
Observed spatially averaged tropospheric NO_2_, SO_2_, and O_3_ column densities. The grey band indicates the lockdown period.

**Fig. 3:**
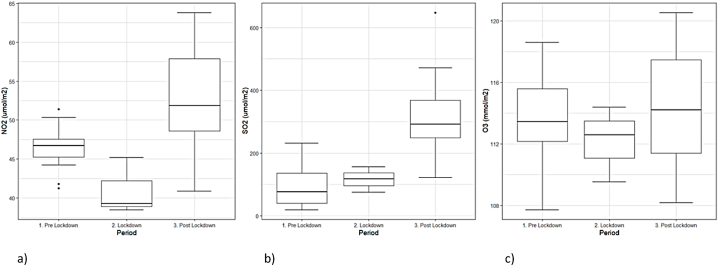
Comparison of column densities for the period before the lockdown, during, and after the lockdown. a) NO_2_; b) SO_2_; c) O_3_.

Monthly mean TVCD values for NO_2_ during the study period (Fig. 2, Table 2) suggest that during the first months after lockdown NO_2_ concentrations increased significantly (as indicated by the significant homogeneity tests; F (2.34) = 8.8, p-value <0.0001; see details in SM) in the study provinces (Fig. 3, Table 2). NO_2_ densities increased from between 42 and 54 μmol/m^2^ during the pre-lockdown and lockdown periods, depending on the province, to 48–62 μmol/m^2^ after lockdown (Table 2). The highest increase was observed in DMQ, with a +19% increase, and Santo Domingo Tsáchilas and Guayas followed closely, with 14% and 15%, respectively.

Satellite images corresponding to monthly mean SO_2_ values (Fig. 1 SM) showed irregular variations in SO_2_ concentrations among the different study areas for the different months within the study period. In the Santa Elena province, an upward trend in SO_2_ concentrations was observed during the study period. In the DMQ, there were no significant monthly variations in SO_2_ concentrations, and a significant increase (p < 0.05) in SO_2_ concentrations was only observed in the post-lockdown period.

On the other hand, in the Morona Santiago province (Table 2), SO_2_ concentrations did not show any significant variations (p > 0.05) during lockdown, while post-lockdown SO_2_ concentrations were significantly lower (p < 0.05). Similarly, SO_2_ concentrations in the Chimborazo province did not experience any significant variations during the lockdown period, while they significantly increased after the lockdown (p < 0.05). In the Guayas province, the lowest concentrations were observed in December 2019 and in the months of March and May 2020. SO_2_ concentrations did not vary significantly during lockdown (p > 0.05); however, after lockdown, SO_2_ concentrations increased significantly (p < 0.05). On the other hand, comparing sulfur dioxide concentrations among all four study provinces and the Metropolitan District of Quito, SO_2_ concentrations in the DMQ were found to be significantly lower than in the other study areas (p < 0.05), and the significantly highest SO_2_ concentrations (p < 0.05) were found in the Chimborazo province; only before lockdown were concentrations in the Morona Santiago province similar to those in Chimborazo (p > 0.05).

When compared to pre-lockdown conditions, average NO_2_ densities after lockdown increased approximately sevenfold, from values close to 50 μmol/m^2^ to around 300 μmol/m^2^, although with a higher variability (Fig. 2). The October higher concentrations may be due to increased traffic with the beginning of school activities and some and in December due to the Christmas holidays. Both indicating a preference for individual transport in the aftermath of the confinement period.

Regarding monthly variations in ozone concentrations during the study period (Fig. 2 SM), a decrease in these concentrations was observed in all sites in Ecuador during the months of April and May. However, when comparing monthly means for ozone among the three study provinces and the Metropolitan District of Quito for the three periods: before, during, and after lockdown (Table 2), no significant increases in concentrations were observed during the whole study period (p > 0.05).

Ozone concentrations in the Metropolitan District of Quito were significantly lower (p < 0.05) than in the provinces of Guayas, Sucumbíos, and Pastaza. Likewise, in the Guayas province, concentrations before and during lockdown were shown to be significantly higher (p < 0.05) than in the Sucumbíos and Pastaza provinces and in the Metropolitan District of Quito; however, when comparing post-lockdown ozone concentrations, the Sucumbíos province showed the highest concentrations (p < 0.05) compared to Guayas, Pastaza, and the Metropolitan District of Quito. On average, ozone concentrations and densities remained constant during the analyzed period (Table 2, Fig. 2).

In Ecuador, there are only two defined seasons: wet or winter and dry or summer [29]. The three regions that conform the continental country have different climates. In the coastal region, the rainy season starts in December and lasts until May; the dry season takes place in June and November with temperature variations between 2 and 3 °C. In the Sierra or Andean region, the rainy season lasts from October to May, and the dry season from June to September, with mean temperatures about 14.5 °C. In the Amazon region there are differences between north and south. In the north Amazon (Sucumbíos province), the rainy season lasts from March to November while the dry season lasts from December to February. In the rest of the Amazon region, the seasonal trend is like the Andean Region with a mean temperature around 21 °C [30]. Furthermore, the location of Ecuador, on the Ecuadorian line, produces a little seasonality throughout the year and the country's mean temperatures have small variation during the whole year [28].

In addition, we examined the correlation between each pollutant and the meteorological parameters for monthly average of air temperature to 1 km height and average of precipitation (8235 records). Weak negative and positive correlations were observed between the meteorological parameters and the trace concentrations of the study gases, the absolute values of these correlations varied from 0.1 to 0.4. Only in the case of the relationship between NO_2_ vertical column density and precipitation a negative correlation of 0.56 was obtained. Hence, this result is indicating the main contribution of precipitation in decreasing air pollution.

## 4 Discussion

Mean NO_2_ concentrations were higher in Guayas than in the remaining provinces; this can be attributed to the fact that its capital, Guayaquil, is the most populated and industrialized city in Ecuador [12]. NO_2_ concentrations decreased during lockdown in all study areas (DMQ: 26%, Guayas: 23%, and Santo Domingo de los Tsáchilas: 12%) and increased again after lockdown:+23% in two of the study areas (DMQ and Santo Domingo de los Tsáchilas); and +28% in the Guayas province. The TROPOMI data of NO2, from versions v1.2 and v.1.3 lead to low tropospheric VCD data by up to 22–51% in polluted áreas [19]. This bias is greatly reduced in version v1.4 from November 29, 2020, which could contribute to high percentages after the confinement. Results similar to ours were obtained by previous studies in Ecuador and in other regions around the world, having been attributed to a shift for private transportation due to health concerns. Thus, a 13% decrease was observed in Ecuador [12], a 50% decrease was found in India [14], and a 50% decrease was recorded in Madrid, Spain [15], while NO_2_ concentrations dramatically decreased in three metropolitan areas in China: Beijing, Wuhan, and Guangzhou [31]. Decreases between 18% and 40% were found in major urban areas of Europe (Madrid, Milan, and Paris) and the United States (New York, Boston, and Springfield). On the other hand, urban areas that were only subjected to partial or inexistent lockdown measures (Warsaw, Pierre, Bismarck, and Lincoln) showed a relatively smaller decrease in mean NO_2_ concentrations (3%–7.5%) [32].

Conversely, ozone showed an anomalous behaviour, since concentrations increased by 2–3% in the study areas despite the decrease in NO_2_, one of its precursor compounds. Previous studies have shown that increased O_3_ concentrations are explained by the lockdown-induced decrease in NOx concentrations, according to reactions 1 to 5 [15]:

(1) $$NO_{2}+h\gamma \to NO+O$$

(2) $$O+O_{2}+M\;\to \;O_{3}+M$$

(3) $$NO+O_{3}\;\to NO_{2}+O$$

(4) $$VOCs+OH\;\to RO_{2}+H_{2}O$$

(5) $$RO_{2}+NO\;\to \;RO+NO_{2}$$

Eq. (3) indicates ozone consumption by NO_x_: when NO_x_ concentration decreases, ozone concentration increases [11,15,30,33].

On the other hand, ozone formation also depends on the concentrations of atmospheric pollutants such as volatile organic compounds (VOCs). Oxidation of VOCs with hydroxyl radicals (OH.) promotes the formation of organic radicals (RO2. and RO.) and ozone through photolysis of NO_2_. NO_2_ is photolyzed to generate atomic oxygen, which then combines with oxygen to generate ozone (Eq. (1)), restarting the described cycle [16]. However, when VOC concentrations remain constant or do not decrease significantly, ozone concentrations increase when the available NOx is insufficient to consume ozone [11,31].

**Fig. 4:**
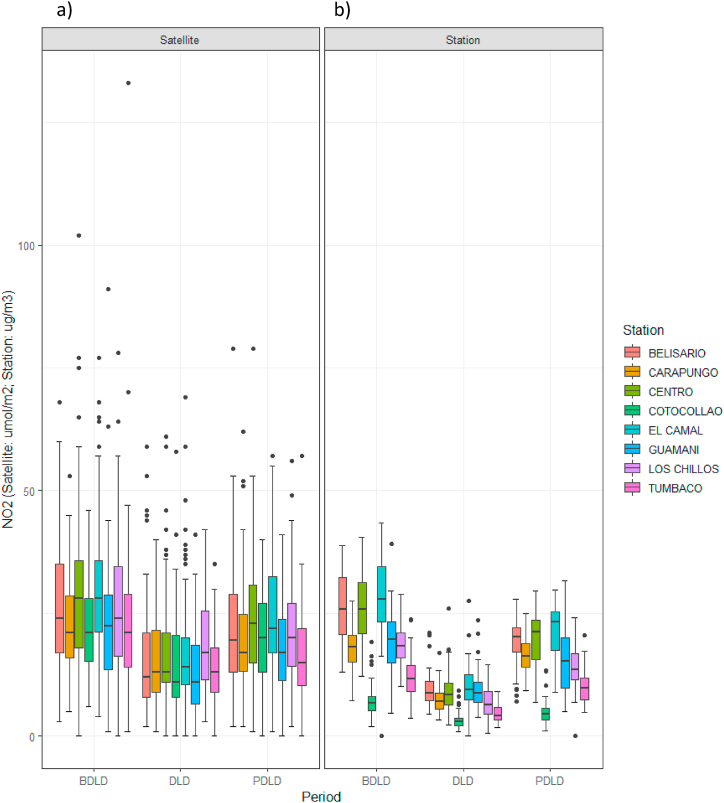
NO_2_ concentrations in the before (BLD), during (LD), and after lockdown (ALD) periods in the DMQ: a) satellite data, b) surface data from monitoring stations.

As for SO_2_, the provinces with the highest total column SO_2_ values were Morona Santiago, Chimborazo, Guayas, and Santa Elena. SO_2_ showed non-significant irregular variations during the lockdown period (Fig. 4 SM); however, an increase was observed after lockdown, which could possibly be partly explained by the eruptive activity of the Sangay volcano, located in the upper eastern flank of the Eastern Cordillera of Ecuador, during 2019–2020 [33]. The emissions released by this major eruptive event were transported towards the southwest, affecting the provinces of Chimborazo, Guayas, Santa Elena, and Morona Santiago [34,35]. Sangay eruptions began in June 2020, the volcano is located in the province of Morona Santiago, the winds generally go from east to west, so the provinces most affected in sequence are Chimborazo, Guayas and Santa Elena, so in Chimborazo concentration increases in June, but the highest concentrations in Guayas and Santa Elena are in July. The second eruption in September, and in October we have the highest concentrations in Chimborazo, Guayas and Santa Elena in that order.

With respect to the results from data validation and comparison, specifically in the case of NO_2_, numerous studies have related column density data to surface concentrations [15,16,18,31,32,36] due to the fact that NO_2_ has a mainly anthropogenic origin and a short lifespan [18]. In this study, when NO_2_ concentrations from satellite data were related to the corresponding data from DMQ monitoring stations, similar variations in concentrations were observed during the study period (Fig. 4a and b).

**Fig. 5:**
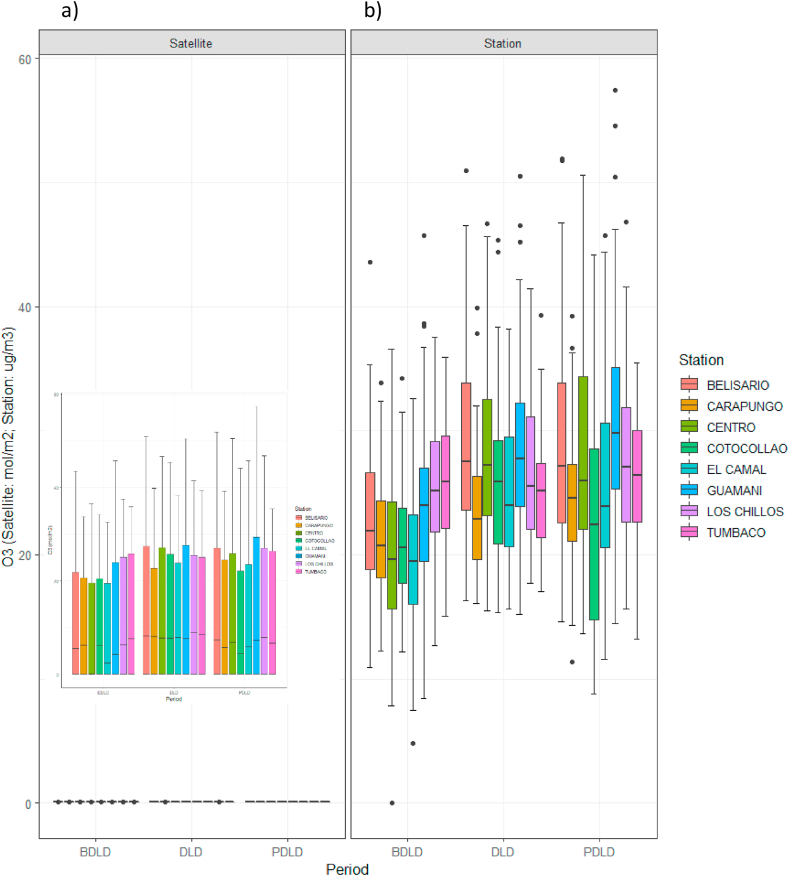
O_3_ concentrations in the before (BLD), during (LD), and after lockdown (ALD) periods in the DMQ: a) satellite data, b) surface data from monitoring stations.

In the case of ozone, studies relating total column density of O_3_ to surface concentrations are scarce [18]; nevertheless, when surface concentrations from DMQ monitoring stations were related to the corresponding satellite data, similar trends in variations were observed (Fig. 5a and b). Moreover, when total column ozone densities in Ecuador (both monthly and for the study period) were related to tropospheric column ozone densities (for which the study area in Ecuador covered only four pixels, since the available resolution was 111320, 55660 m), the same upward trend was observed in the two data series (Fig. 5 SM).

Additionally, validation of satellite data by linear regression of level-3 data for NO_2_, O_3_, and SO_2_ concentrations against the corresponding data from DMQ monitoring stations (Table 2 SM) showed a regression coefficient higher than r = 0.9 for ozone concentrations, while the mean regression coefficient was 0.7 for NO_2_ and 0.24 for SO_2_. Similar studies found similar correlations for NO_2_ (R^2^ = 0.72) [14]; likewise, no significant correlations were found for SO_2_ [36]. Despite the good correlation between satellite and ground measurements for ozone, its modelling is challenging as its changes are determined by complex, interdependent interactions between precursor emissions, atmospheric transport, photochemical production, deposition, exchange between stratosphere-troposphere, and by the fact that ozone columns measure mainly the contribution of non-tropospherice ozone. Still, the contribution of tropospheric ozone has been shown to be detectable and quantifiable [37]. As far as monthly climatological records are concerned, the year 2020 can be considered within the average (Fig. 6 SM and reference [38]), so weather conditions do not seem to have been in the origin of the detected alterations.

## 5 Conclusions

The pollutants analyzed in this study experienced a general decrease during the lockdown caused by the Covid-19 pandemic; however, not all of them exhibited the same pattern. NO_2_ was the pollutant that experienced the highest decrease associated with lockdown, due to reduced human activity, but returned to values close or above to those of the pre-confinement period. Atmospheric SO_2_ concentrations in Ecuador are strongly related to geological processes, particularly with volcanic activity, while the increase in O_3_ concentrations during lockdown can be explained because its concentration in the lower layers of the atmosphere depends on multiple reactions and processes, especially on the reduction of nitrogen oxides.

## Author contribution statement

Oliva Atiaga: conceived and designed the experiments, analyzed and interpreted the data, performed the experiments, wrote the paper.

Fernanda Guerrero, Fernando Páez, Rafael Castro, Edison Collahuazo, Marcelo Grijalva, and Iván Grijalva: analyzed and interpreted the data, wrote the paper.

Luís Miguel Nunes; Xosé Luis Otero : analyzed and interpreted the data, wrote the paper.

## Data availability statement

Data will be made available on request.

## Declaration of competing interest

The authors declare the following financial interests/personal relationships which may be considered as potential competing interests: This study is part of a research project funded by the Universidad de las Fuerzas Armadas-ESPE through Project 2021-PIC-001-CTE and Xunta de Galicia-Consellería de Educacion e Ordeanción Universitaria de Galicia (Consolidation of competitive groups of investigation; GRC GI 1574) and the CRETUS strategic group (AGRUP2015/02).

## References

[1] Rovira J., Domingo J.L., Schuhmacher M. (2020). Air quality, health impacts and burden of disease due to air pollution (PM10, PM2.5, NO_2_ and O_3_): application of AirQ+ model to the Camp de Tarragona County (Catalonia, Spain). Sci. Total Environ..

[2] Cheung C.W., He G., Pan Y. (2020). Mitigating the air pollution effect? The remarkable decline in the pollution-mortality relationship in Hong Kong. J. Environ. Econ. Manag..

[3] Ebenstein A., Fan M., Greenstone M., He G., Zhou M. (2017). New evidence on the impact of sustained exposure to air pollution on life expectancy from China's Huai River Policy. Proc. Natl. Acad. Sci. U.S.A..

[4] Who (2016).

[5] Schikowski T., Altuğ H. (2020). The role of air pollution in cognitive impairment and decline. Neurochem. Int..

[6] European Environment Agency (2019).

[7] Valencia V.H., Hertel O., Ketzel M., Levin G. (2020). Modeling urban background air pollution in Quito, Ecuador. Atmos. Pollut. Res..

[8] Peña Murillo S.E. (2018). Impacto de la contaminación atmosférica en dos principales ciudades del Ecuador. Revista Universidad y Sociedad.

[9] Mancheno T., Zalakeviciute R., González-Rodríguez M., Alexandrino K. (2021). Assessment of metals in PM10 filters and Araucaria heterophylla needles in two areas of Quito, Ecuador. Heliyon.

[10] Comité de operaciones de Emergencia Nacional (COE) (2020).

[11] Cazorla M., Herrera E., Palomeque E., Saud N. (2021). What the COVID-19 lockdown revealed about photochemistry and ozone production in Quito, Ecuador. Atmos. Pollut. Res..

[12] Pacheco H., Díaz-López S., Jarre E., Pacheco H., Méndez W., Zamora-Ledezma E. (2020). NO2 levels after the COVID-19 lockdown in Ecuador: a trade-off between environment and human health. Urban Clim..

[13] Comité de operaciones de Emergencia Nacional (COE) (2020).

[14] Zheng Z., Yang Z., Wu Z., Marinello F. (2019). Spatial variation of NO_2_ and its impact factors in China: an application of sentinel-5P products. Rem. Sens..

[15] Rathod A., Sahu S.K., Singh S., Beig G. (2021). Anomalous behaviour of ozone under COVID-19 and explicit diagnosis of O_3_-NO_x_-VOCs mechanism. Heliyon.

[16] Baldasano J.M. (2020). COVID-19 lockdown effects on air quality by NO2 in the cities of Barcelona and Madrid (Spain). Sci. Total Environ..

[17] Ogen Y. (2020). Assessing nitrogen dioxide (NO2) levels as a contributing factor to coronavirus (COVID-19) fatality. Sci. Total Environ..

[18] Kang Y., Choi H., Im J., Park S., Shin M., Song C. (2021). Estimation of surface-level NO2 and O3 concentrations using TROPOMI data and machine learning over East Asia. Environ. Pollut..

[19] Van Geffen J., Eskes H., Compernolle S., Pinardi G., Verhoelst T., Lambert J.- (2020). Sentinel-5P TROPOMI NO2 retrieval: impact of version v2.2 improvements and comparisons with OMI and ground-based data. Atmos Meas. Tech. Sci. Total Environ..

[20] Lerot C., Heue K.-P., Verhoelst T., Lambert J.-C., Balis D., Garane K., Granville G., Koukouli M.E., Loyola D., Romahn F., Van Roozendael M., Xu J., Zimmer W., Bazureau A., Fioletov V., Goutail F., McLinden C., Pazmiño A., Pommereau J.-P. (2018). https://sentinel.esa.int/documents/247904/3541451/Sentinel-5P-Readme-OFFL-Total-Ozone.pdf.

[21] Theys N., Hedelt P., De Smedt I., Lerot C., Yu H., Vlietinck J., Pedergnana M., Arellano S., Galle B., Fernandez D., Carlito C.J.M., Barrington C., Taisne B., Delgado-Granados H., Loyola D., Van Roozendael M. (2019). Global monitoring of volcanic SO2 degassing with unprecedented resolution from TROPOMI onboard Sentinel-5 Precursor. Sci. Rep..

[22] Crosman E. (2021). Meteorological drivers of permian basin methane anomalies derived from TROPOMI. Rem. Sens..

[23] Shikwambana L., Mhangara P., Mbatha N. (2020). Trend analysis and first time observations of sulphur dioxide and nitrogen dioxide in South Africa using TROPOMI/Sentinel-5 P data. Int. J. Appl. Earth Obs. Geoinf..

[24] (2017). Earth Engine Data Catalog, Sentinel Collections.

[25] Alvarez-Mendoza C.I., Teodoro A., Freitas A., Fonseca J. (2020). Spatial estimation of chronic respiratory diseases based on machine learning procedures—an approach using remote sensing data and environmental variables in Quito, Ecuador. Appl. Geogr..

[26] Veefkind J.P., Aben I., McMullan K., Förster H., de Vries J., Otter G. (2012). TROPOMI on the ESA Sentinel-5 Precursor: a GMES mission for global observations of the atmospheric composition for climate, air quality and ozone layer applications. Remote Sens. Environ..

[27] Ramírez L., Falcón V. (2015).

[28] Fisher R.A., Kotz S., Johnson N.L. (2022). Breakthroughs in Statistics.

[29] Ron S.R., Merino-Viteri A., Ortiz D.A. (2022). Anfibios del Ecuador. Museo de Zoología, Pontificia Universidad Católica del Ecuador. https://bioweb.bio/faunaweb/amphibiaweb.

[30] (2023). Climate change knowledge portal of the world bank (CCKP). https://climateknowledgeportal.worldbank.org/.

[31] Pei Z., Han G., Ma X., Su H., Gong W. (2020). Response of major air pollutants to COVID-19 lockdowns in China. Sci. Total Environ..

[32] Bar S., Parida B.R., Mandal S.P., Pandey A.C., Kumar N., Mishra B. (2021). Impacts of partial to complete COVID-19 lockdown on NO2 and PM2.5 levels in major urban cities of Europe and USA. Cities.

[33] Zhao F., Liu C., Cai Z., Liu X., Bak J., Kim J. (2021). Ozone profile retrievals from TROPOMI: implication for the variation of tropospheric ozone during the outbreak of COVID-19 in China. Sci. Total Environ..

[34] Valverde V., Mothes P.A., Beate B., Bernard J. (2021). Enormous and far-reaching debris avalanche deposits from Sangay volcano (Ecuador): multidisciplinary study and modeling the 30 ka sector collapse. J. Volcanol. Geoth. Res..

[35] Instituto Geofísico. Escuela Politécnica Nacional. Informe Especial del Volcán Sangay No 1-2020. Quito, Ecuador. https://www.igepn.edu.ec/servicios/noticias/1816-informe-especial-del-volcan-sangay-n-1-2020.

[36] Ghasempour F., Sekertekin A., Kutoglu S.H. (2021). Google Earth Engine based spatio-temporal analysis of air pollutants before and during the first wave COVID-19 outbreak over Turkey via remote sensing. J. Clean. Prod..

[37] Ziemke J.R., Oman L.D., Strode S.A., Douglass A.R., Olsen M.A., McPeters R.D., Bhartia P.K., Froidevaux L., Labow G.J., Witte J.C., Thompson A.M., Haffner D.P., Kramarova N.A., Frith S.M., Huang L.-K., Jaross G.R., Seftor C.J., Deland M.T., Taylor S.L. (2019). Trends in global tropospheric ozone inferred from a composite record of TOMS/OMI/MLS/OMPS satellite measurements and the MERRA-2 GMI simulation. Atmos. Chem. Phys..

[38] EPMAPS. Anuario Hidrometeorológico (2020).

