# Supplementary material for: Assessment of variations in air quality in cities of Ecuador in relation to the lockdown due to the COVID-19 pandemic
Source: Heliyon. 2023 Jun 12;9(6):e17033. doi: 10.1016/j.heliyon.2023.e17033 (PMC10361106; doi:10.1016/j.heliyon.2023.e17033)
Supplement: Multimedia component 1 [file mmc1.docx]

Table 1 SM. Description and characteristics of the gas and particle analyzers of the REMMAQ

| Pollutant | Number | Location | Measurement method or principle of operation | Brand and model |
| --- | --- | --- | --- | --- |
| Particulate Matter PM_10_ | 4 | Tumbaco, Guamaní,Carapungo, SAP | Beta Ray Attenuation (EPA PM10 Equivalent Method No. EQPM-1102-150) | Thermo Scientific/FH62C14 |
| Particulate Matter PM2.5 | 6 | Belisario, Camal,Centro,Cotocollao, Carapungo, SAP | Atenuación de rayos beta (Método equivalente para PM10 EPA No. EQPM-1102-150) | Thermo Andersen / FH62C14 |
| Sulphur dioxide | 7 | Belisario, Camal,Centro, Tumbaco, Cotocollao, Carapungo, Chillogallo | Pulsed Ultraviolet Light Fluorescence (EPA Equivalent Method No. EQSA-0486-060) | THERMO 43C / 43i |
| Ozone | 10 | Belisario, Camal,Centro, Tumbaco, Chillogallo, Cotocollao, Carapungo, Guamaní, Jipijapa,Electronic Lab. | Ultraviolet light absorption (EPA equivalent Method No. EQOA-0880-047) | THERMO 49C / 49i |
| Nitrogen dioxide | 8 | Belisario, Camal,Centro, Cotocollao, Carapungo, Guamaní, Jipijapa, Chillogallo | Chemiluminescence EPA Reference Method No. RFNA-1289-074) | THERMO 42C / 42i |
| Carbon monoxide | 8 | Belisario, Camal,Centro, Cotocollao, Carapungo, Guamaní, Jipijapa, Electronic Lab. | Non-dispersive infrared absorption (EPA Reference Method No. RFCA-0981-054) | THERMO / 48C / 48i |
| Multicalibrador | 12 | Belisario, Camal,Centro, Tumbaco, Chillogallo, Cotocollao, Carapungo, Guamaní, Jipijapa, Electronic Lab., movil S. | Principle of operation: Dilution of gases, zero air with a certified reference material (contaminant of known concentration). | THERMO/ 146C / 146i |

**Source:** Municipality of Quito, Environment Secretary, Atmospheric Monitoring Network.2019.

**Table 2 SM.** Conversion coefficients for satellite measurements, based on daily values. Slope is the ratio between concentrations made in measuring stations (REMMAQ) and satellite estimates.

|  | **Gas** | | | | | |
| --- | --- | --- | --- | --- | --- | --- |
|  | **SO_2_** | | **NO_2_** | | **O_3_** | |
| **Station** | **Slope**  **(umol m^3^)/(ug m^2^)** | **R** | **Slope**  **(umol m^3^)/(ug m^2^)** | **R** | **Slope**  **(mol m^3^) /(ug m^2^)** | **R** |
| Belisario | 0.0168 | 0.24 | 0.542 | 0.72 | 240.14 | 0.93 |
| Carapungo | 0.0127 | 0.29 | 0.359 | 0.76 | 208.7 | 0.96 |
| Centro | 0.0154 | 0.27 | 0.381 | 0.67 | 226.3 | 0.92 |
| Cotocollao | 0.0083 | 0.20 | 0.107 | 0.62 | 208.02 | 0.92 |
| El Camal | 0.0186 | 0.27 | 0.544 | 0.76 | 203.65 | 0.92 |
| Guamani | 0.0107 | 0.18 | 0.510 | 0.68 | 249.03 | 0.92 |
| Los Chillos | 0.0166 | 0.21 | 0.425 | 0.63 | 237.89 | 0.96 |
| Tumbaco | 0.0122 | 0.25 | 0.248 | 0.67 | 232.22 | 0.97 |
| AVERAGE | 0.0139 |  | 0.389 |  | 225.74 |  |


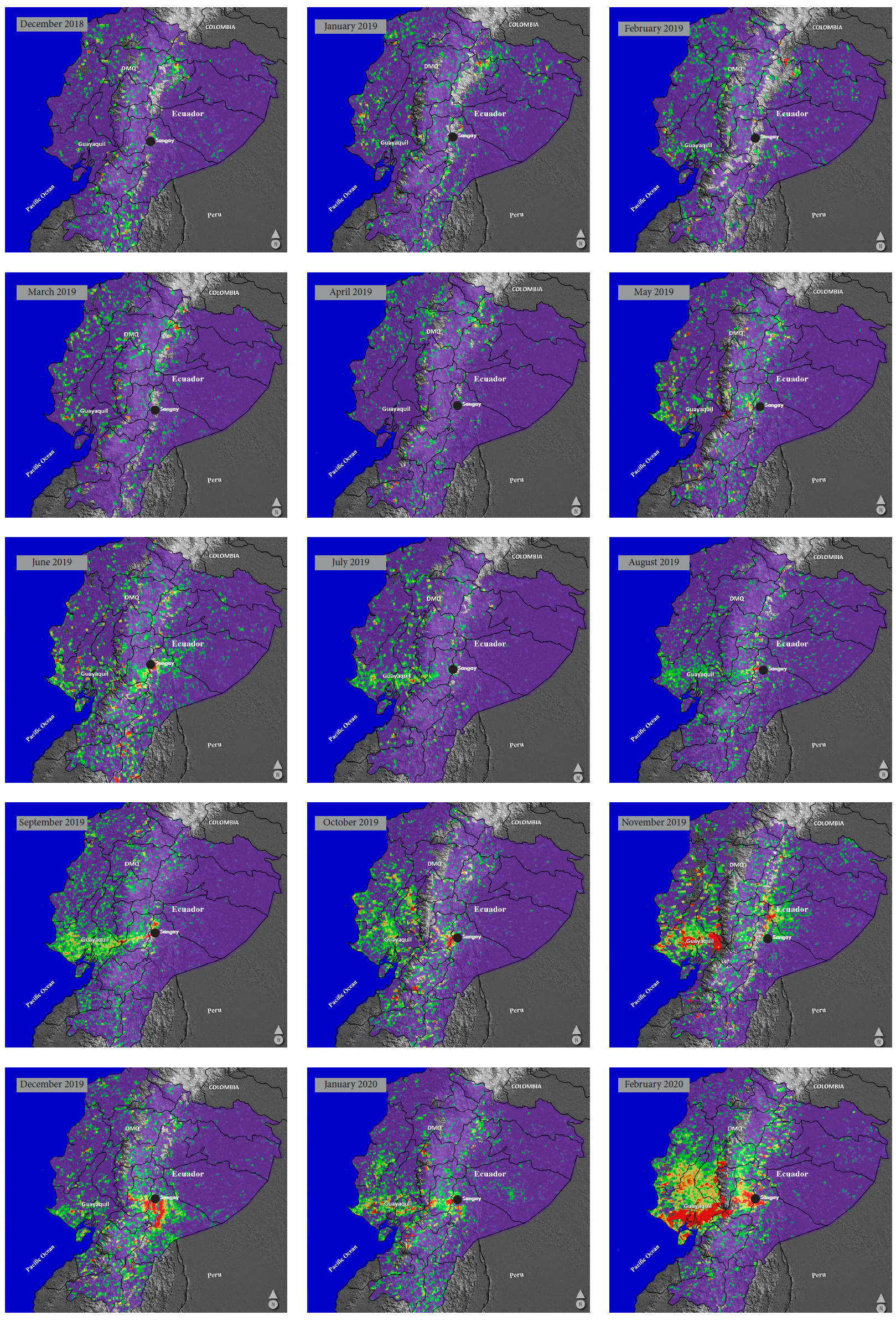


Figure 1 SM. Monthly variation of SO2 concentrations in Ecuador during the period between December 2019 - August 2020


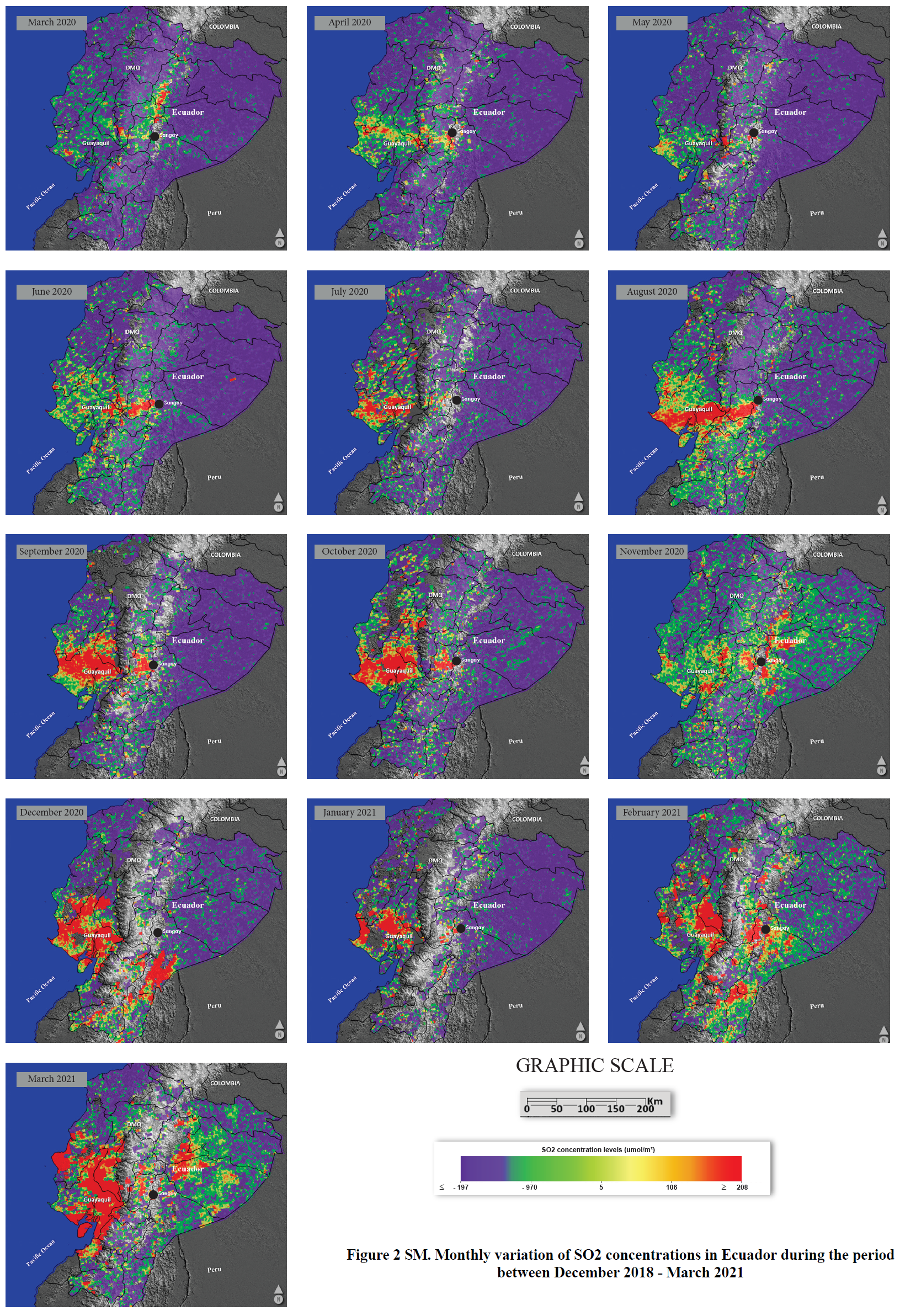


Figure 1 SM (con.). Monthly variation of SO2 concentrations in Ecuador during the period between December 2019 - August 2020


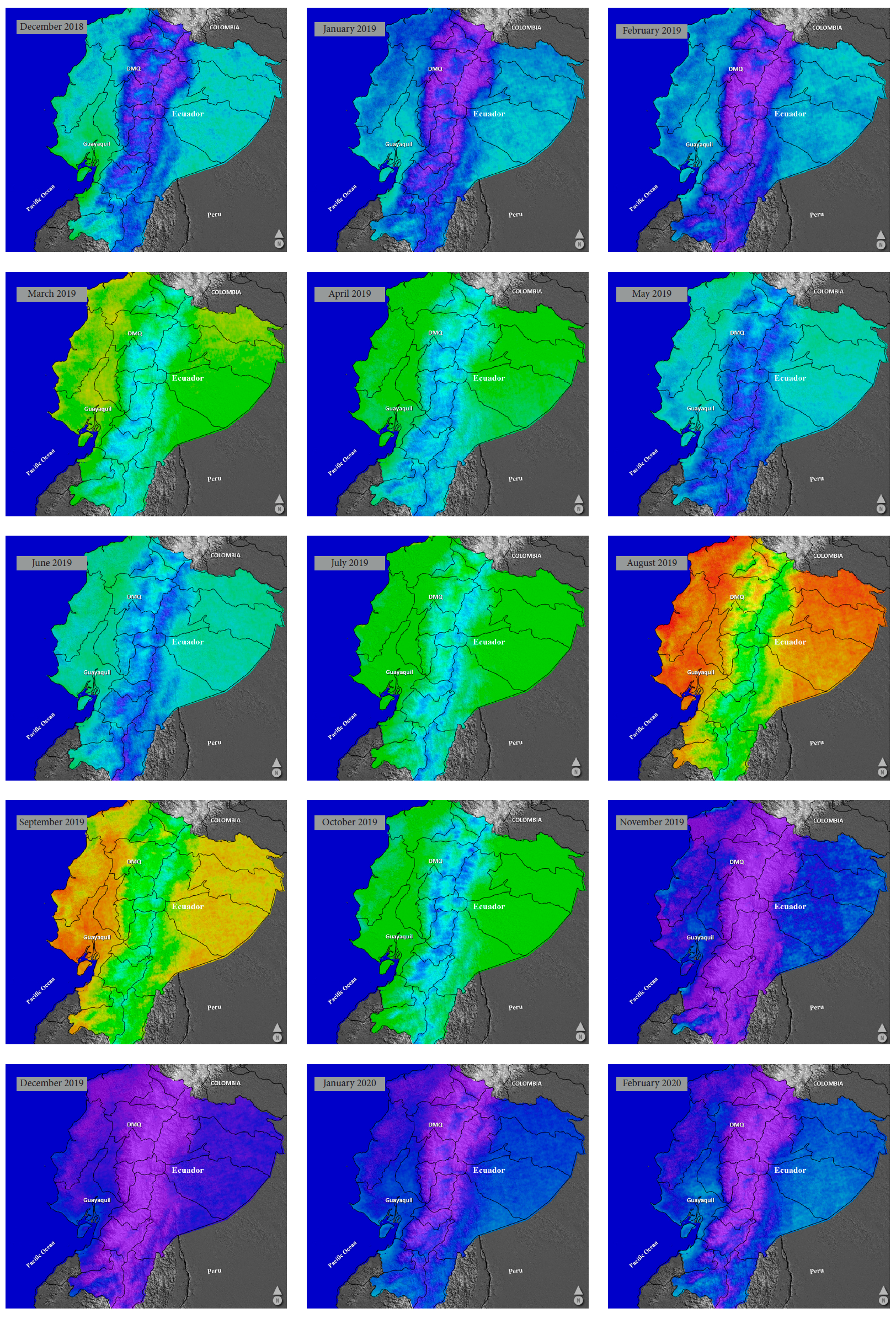


Figure 2 SM. Monthly variation of Ozone concentrations in Ecuador during the period between December 2019 - August 2020


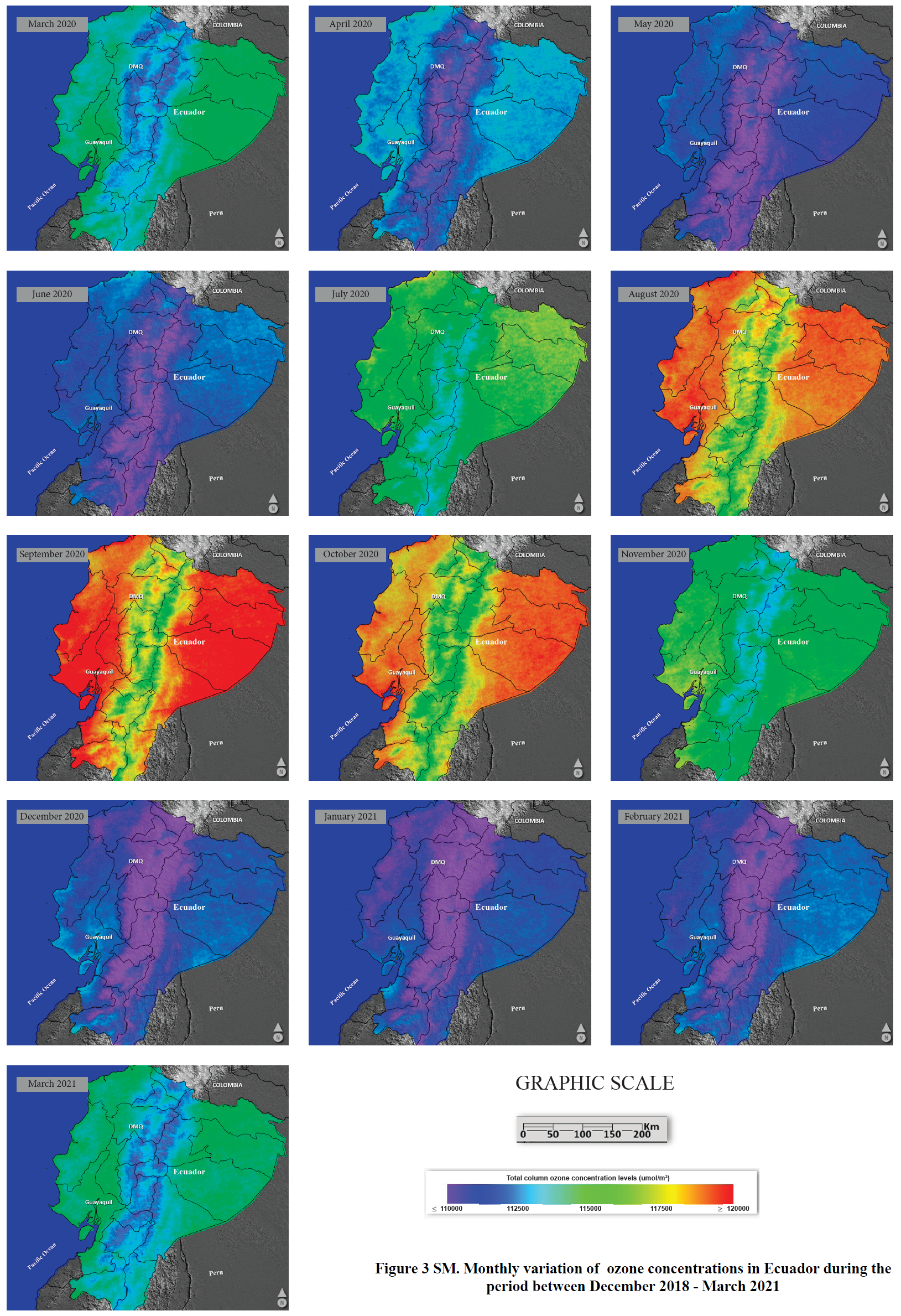


Figure 2 SM (cont.). Monthly variation of Ozone concentrations in Ecuador during the period between December 2019 - August 2020


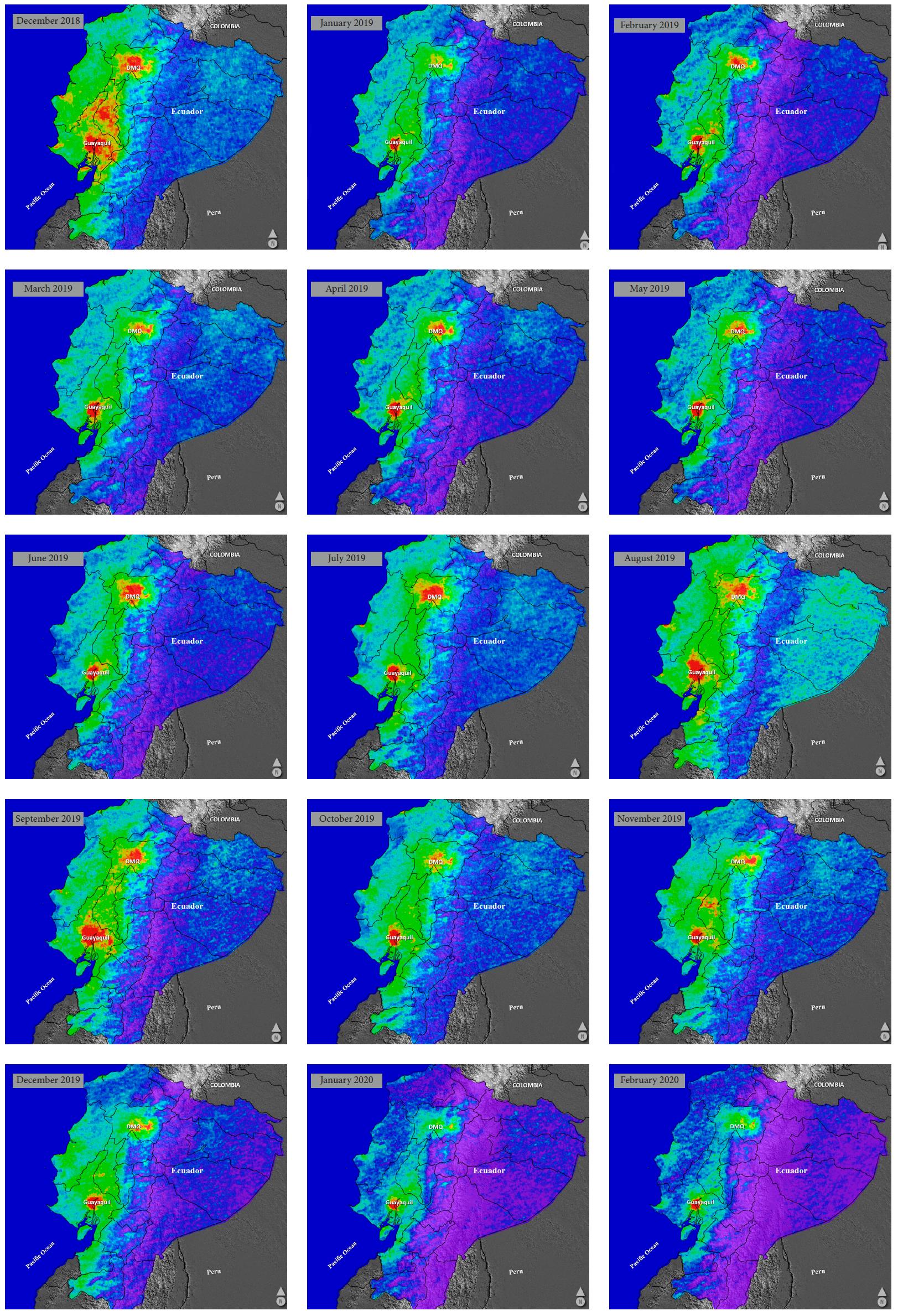
Figure 3 SM. Monthly variation of NO_2_ concentrations in Ecuador during the period between December 2019 - August 2020


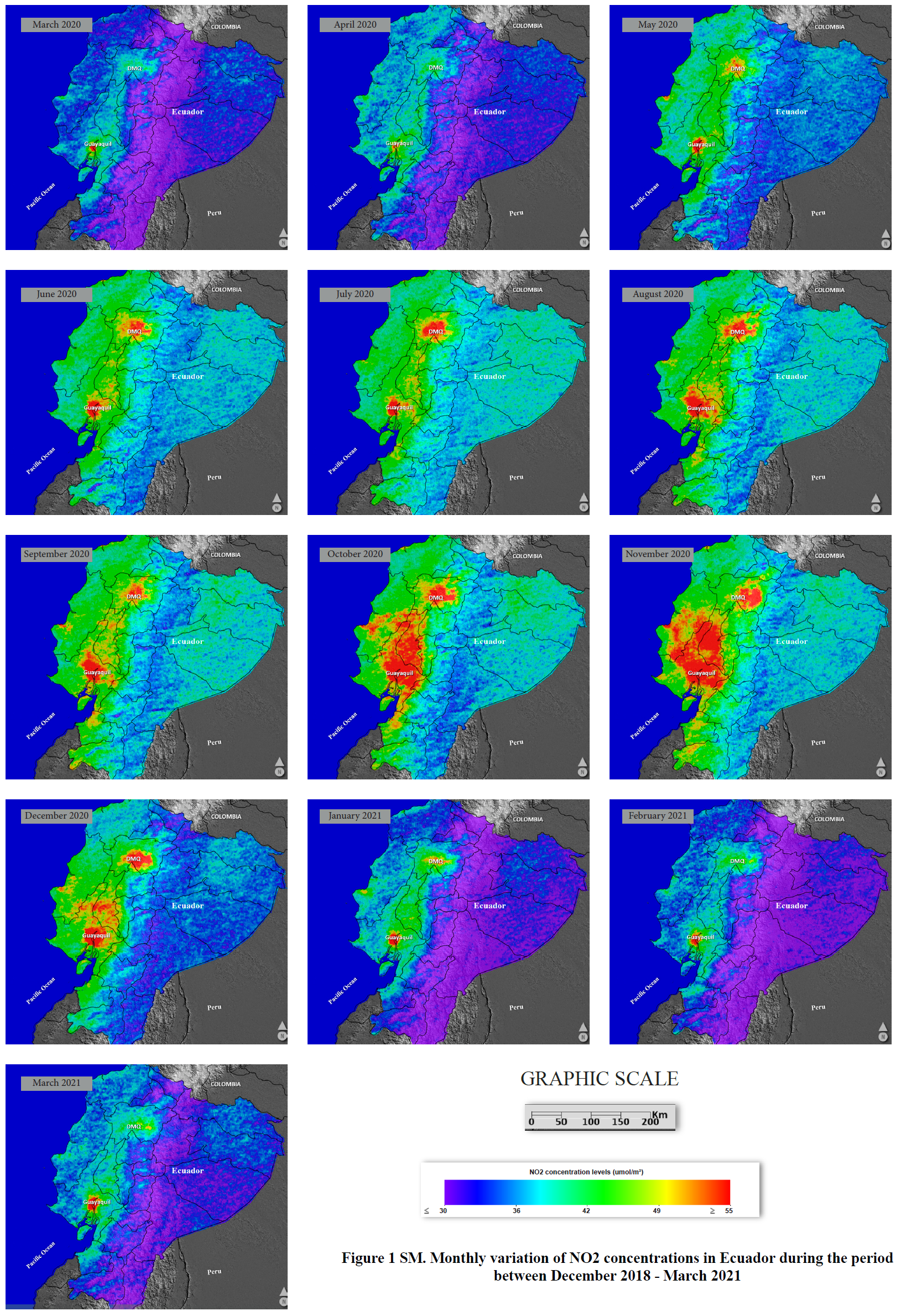


Figure 3 SM (cont.). Monthly variation of NO_2_ concentrations in Ecuador during the period between December 2019 - August 2020

a)


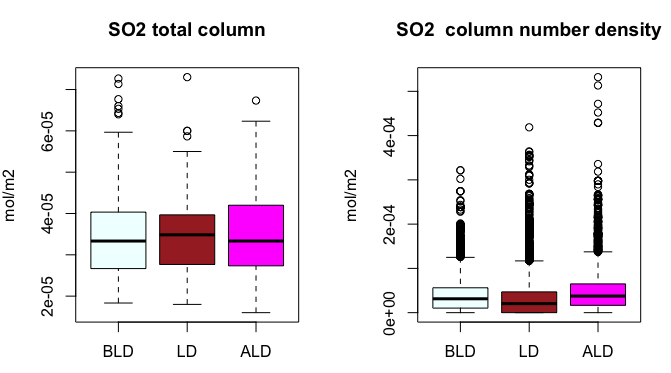


b)


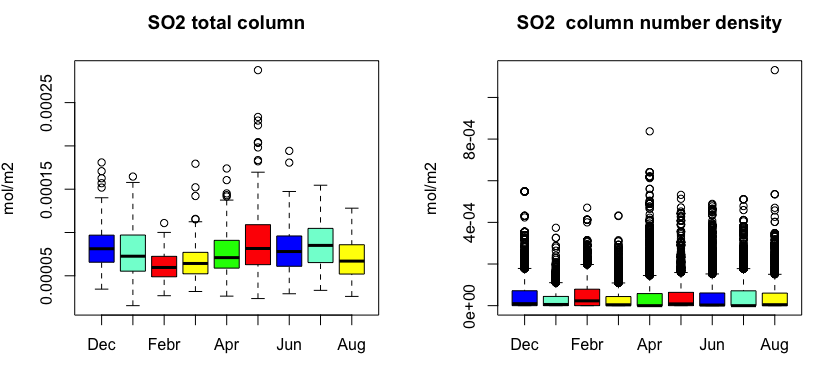


c)

Figure 4 SM. A) SO2 densities in provinces of Ecuador in the before (BLD), during (LD), and after lockdown (ALD) periods; b) SO_2_ total column concentrations and SO_2_ column number density concentrations during the periods (before lockdown (BLD), Lockdown (LD) and after lockdown (ALD) in DMQ; c) Monthly variation of SO_2_ total column concentrations and SO_2_ column number density concentrations in DMQ during the period December 2019 - August 2020


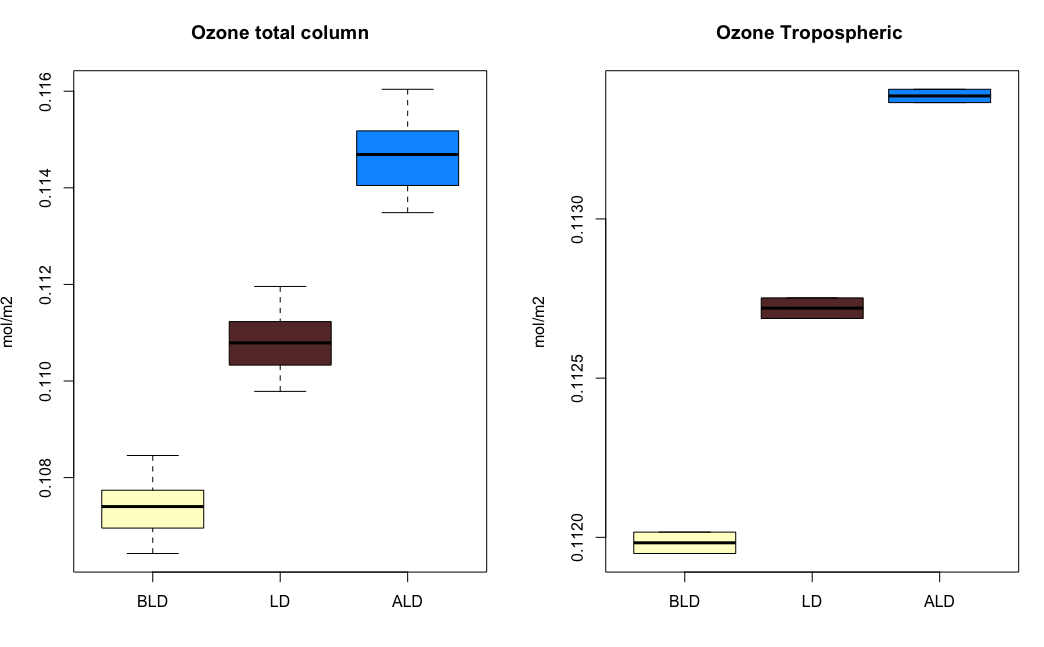


a)


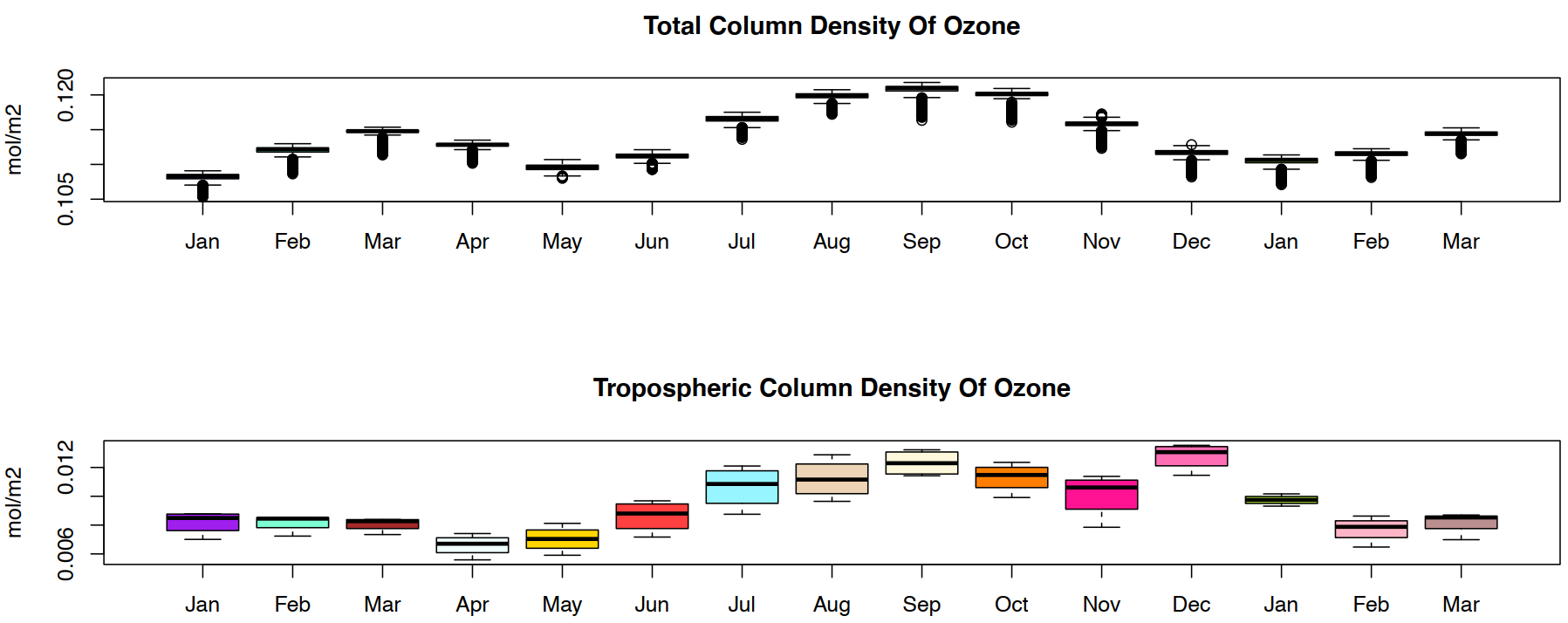


b)

Figure 5 SM. a) Ozone total column concentrations and Ozone Tropospheric Column concentrations during the periods (before lockdown (BLD), Lockdown (LD) and after lockdown (ALD) in DMQ b) Monthly variation of Ozone total column concentrations and Ozone Tropospheric Column concentrations in DMQ during the period December 2019 - August 2020.


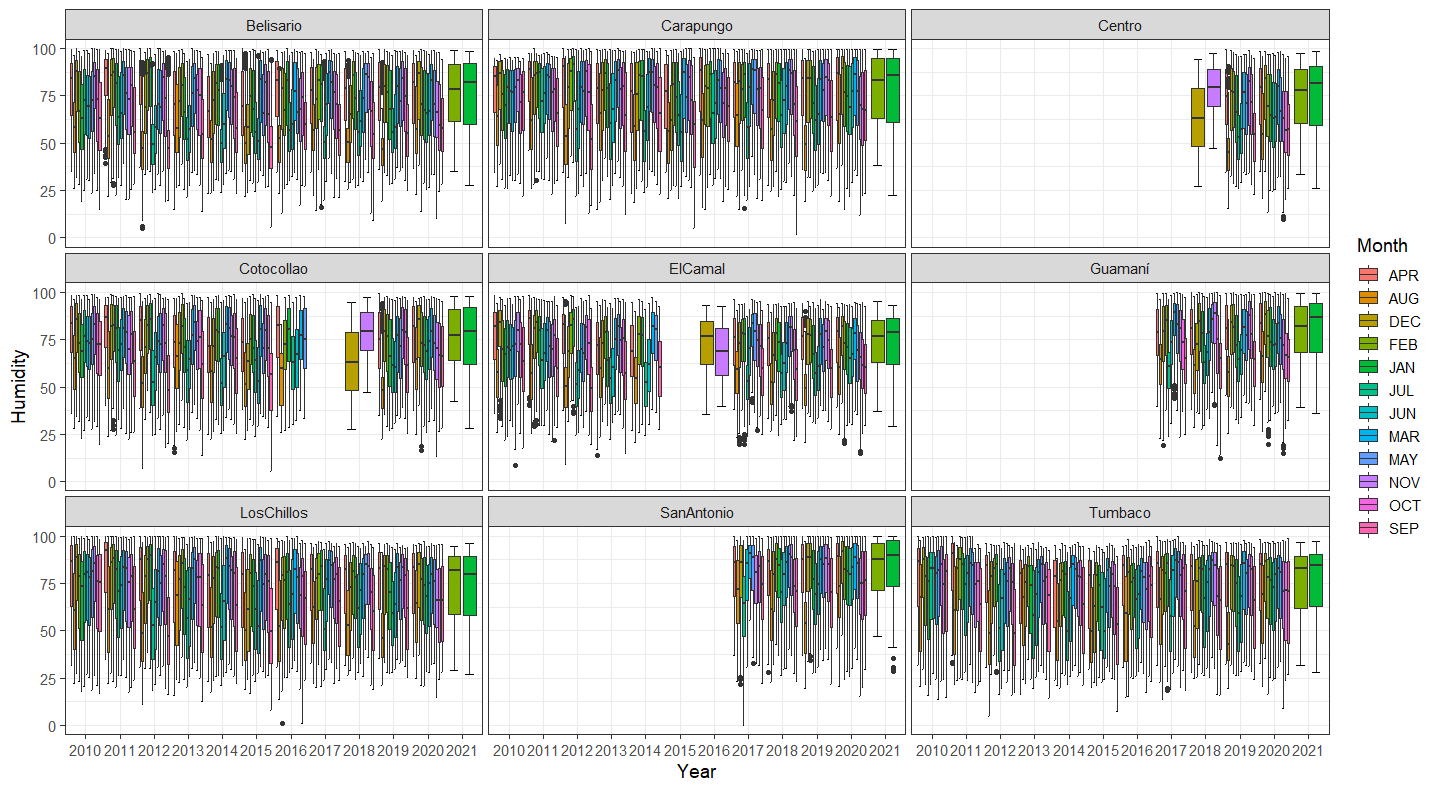


a)


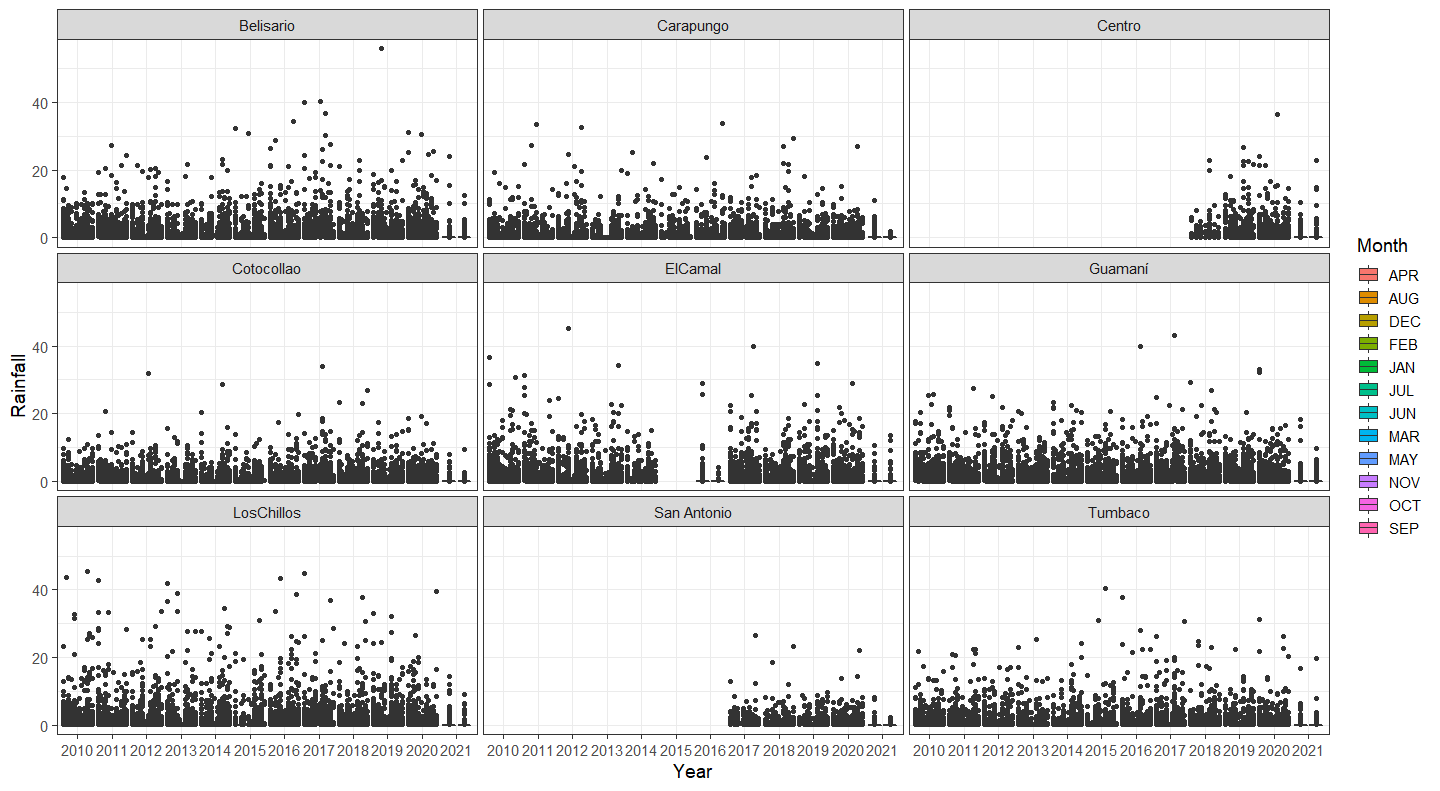


b)


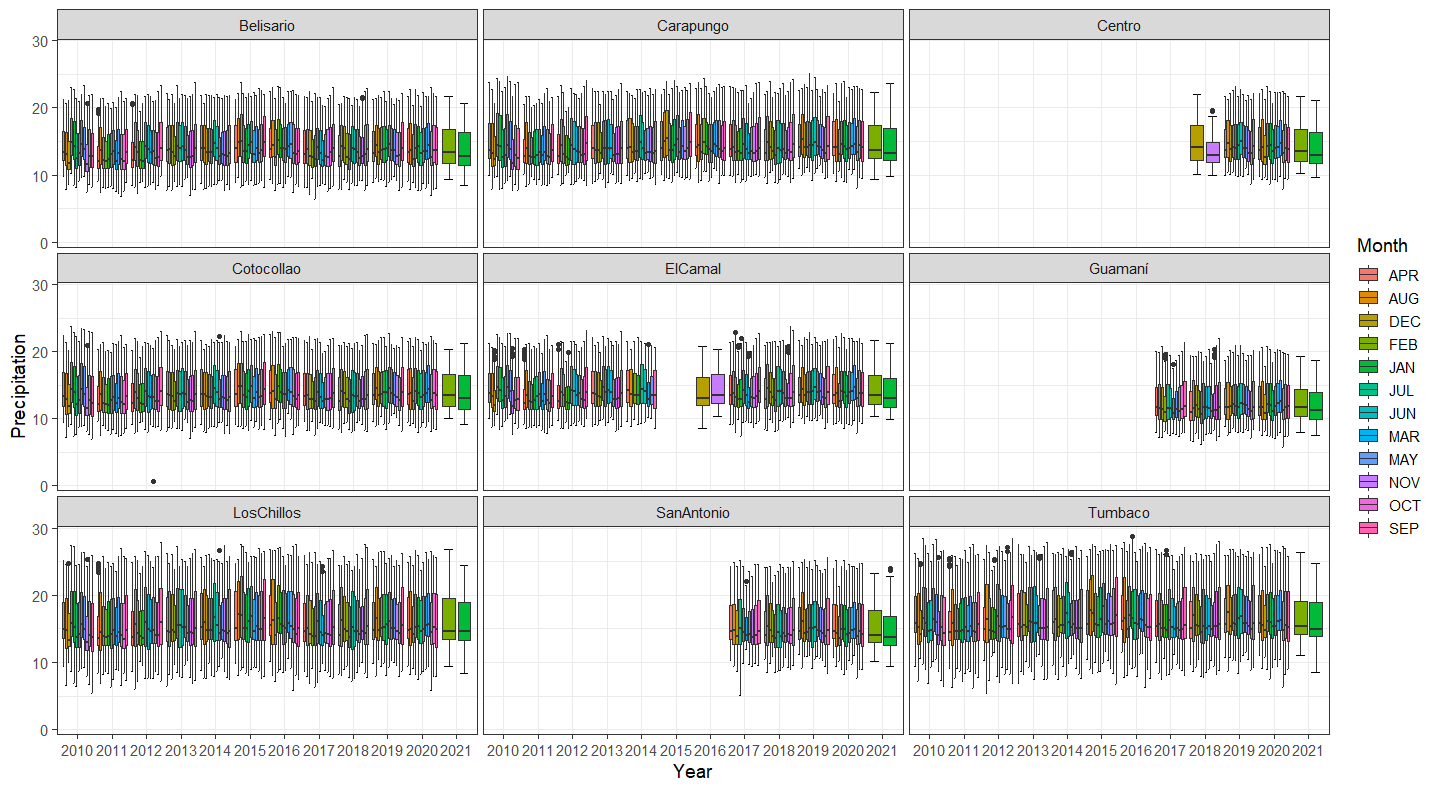


c)

Figure 6 SM. Meteorological in the period 2010-2021 (daily data): a) Humidity (%); b) rainfall (mm); c) temperature (°C)


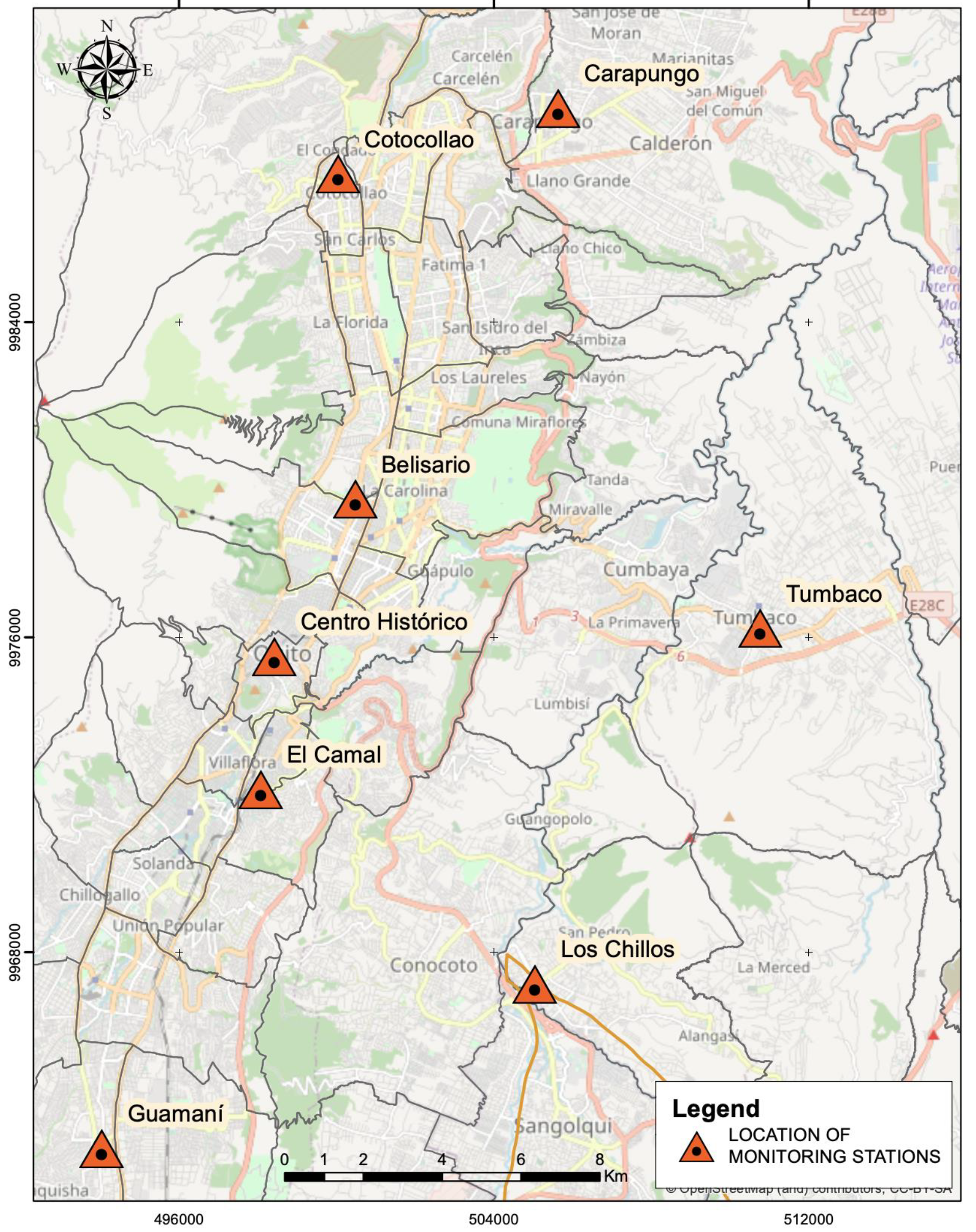


Figure 7 SM. Location of the Monitoring Stations of the REMMAQ
